# Pattern breaking: a complex systems approach to psychedelic medicine

**DOI:** 10.1093/nc/niad017

**Published:** 2023-07-06

**Authors:** Inês Hipólito, Jonas Mago, Fernando E Rosas, Robin Carhart-Harris

**Affiliations:** Berlin School of Mind and Brain, Humboldt-Universität zu Berlin, Berlin 10117, Germany; Department of Philosophy, Macquarie University, New South Wales 2109, Australia; Wellcome Centre for Human Neuroimaging, University College London, London WC1N 3AR, United Kingdom; Integrative Program in Neuroscience, McGill University, Montreal, Quebec QC H3A, Canada; Department of Brain Sciences, Centre for Psychedelic Research, Imperial College London, London SW7 2BX, United Kingdom; Centre for Complexity Science, Imperial College London, London SW7 2BX, United Kingdom; Data Science Institute, Imperial College London, London SW7 2BX, United Kingdom; Department of Informatics, University of Sussex, Brighton BN1 9RH, United Kingdom; Centre for Eudaimonia and Human Flourishing, University of Oxford, Oxford OX3 9BX, United Kingdom; Department of Brain Sciences, Centre for Psychedelic Research, Imperial College London, London SW7 2BX, United Kingdom; Psychedelics Division, University of California San Francisco, San Francisco, CA 92521, United States

**Keywords:** complex systems theory, psychedelics, psychological experience, entropic brain, free-energy principle

## Abstract

Recent research has demonstrated the potential of psychedelic therapy for mental health care. However, the psychological experience underlying its therapeutic effects remains poorly understood. This paper proposes a framework that suggests psychedelics act as destabilizers, both psychologically and neurophysiologically. Drawing on the ‘entropic brain’ hypothesis and the ‘RElaxed Beliefs Under pSychedelics’ model, this paper focuses on the richness of psychological experience. Through a complex systems theory perspective, we suggest that psychedelics destabilize fixed points or attractors, breaking reinforced patterns of thinking and behaving. Our approach explains how psychedelic-induced increases in brain entropy destabilize neurophysiological set points and lead to new conceptualizations of psychedelic psychotherapy. These insights have important implications for risk mitigation and treatment optimization in psychedelic medicine, both during the peak psychedelic experience and during the subacute period of potential recovery.

## Introduction

The increasing societal acceptance and stigmatization of psychedelic (for a classification and characterization of psychedelics, see Kelmendi et al. 2022) substances have opened up novel avenues of scientific inquiry into their potential therapeutic applications for psychopathology (Dos Santos et al. 2020, Marks and Cohen 2021). Emerging experimental data suggest that psychedelics could potentially serve as a transformative new treatment paradigm for psychiatric, social, and palliative care (Byock 2018a, Gründer 2021, Gründer and Jungaberle 2021, Hutchison and Bressi 2021, Enos 2022), albeit with attendant risks and critique (Johnstad 2021, Hall 2022). A salient advantage of psychedelics is their relatively low potential for addiction and physical side effects, rendering them potentially superior to several current pharmacological treatments for mental health care (Read and Williams 2018, Davies et al. 2019, Jauhar et al. 2019, Guy et al. 2020, Lewis 2021).

Recent scientific findings suggest that psychedelic therapy has the potential to generate significant and sustained antidepressant and anxiolytic effects, including for individuals with treatment-resistant depression (Carhart-Harris et al. 2018b, Stroud et al. 2018, Muttoni et al. 2019, Fauvel et al., 2023, Hesselgrave et al. 2021). Additionally, positive outcomes have been observed in cases of high suicidality (Byock, 2018a) and psychological distress associated with a terminal illness (Schimmel et al. 2022). Psychedelic therapy could also prove beneficial in cases of post-traumatic stress disorder (PTSD) (Doblin 2002, Davis et al. 2020, Figueiredo et al. 2021, Rubin-Kahana et al. 2021, Henner et al. 2022), eating disorders (Renelli et al. 2020, Spriggs et al. 2021, Teixeira et al. 2021), obsessive-compulsive disorder (OCD) (Ehrmann et al. 2021), and other common mental health conditions (Krebs and Johansen 2013, Nutt and Carhart-Harris 2021, Simonsson et al. 2021).

A plethora of empirical evidence indicates that the quality of the acute psychedelic experience is a strong predictor of long-term mental health improvements (Letheby 2021, chaps. 2 and 3, Roseman et al. 2019). This suggests that the subjective effects of psychedelics may be crucial for the maintenance of therapeutic outcomes (Yaden and Griffiths 2020, Lutkajtis 2021). Notably, psychedelics often elicit experiences that are perceived as insightful and imbued with profound meaning by participants (Johnson et al. 2019, Hearn 2021, Pedersen et al. 2021). Therefore, therapeutic progress with psychedelics is epistemic or learning based, which could account for its sustained efficacy and failures when the experience falls short. This epistemic dimension of psychedelic therapy underscores the importance of integrating it with a psychologically sophisticated approach, including adept and sufficient psychotherapeutic support (Nayak and Johnson 2021).

A comprehensive biological understanding of psychedelics is imperative, in conjunction with the application of expert psychological knowledge. The former could help circumvent risks associated with their misuse, as well as dispel fallacious philosophical suppositions, such as the notion that they do not exert a fundamental influence on brain function (Timmermann et al. 2021). Additionally, resolving uncertainties surrounding the biological mechanisms of psychedelic action could substantiate their medicinal properties and worth, such as by validating the clinical benefits observed in recent trials as more than just a placebo response (Luoma et al. 2020).

The current study draws from a complex systems theory (CST), which offers a framework that can be applied to both psychological and neurological phenomena by identifying fundamental shared mechanisms at the appropriate dynamical systems level. In this context, we propose that psychedelics act as destabilizers at both the experiential and neurophysiological levels, by influencing set points or ‘attractors’—system-level substates that become stereotyped in psychopathology due to repetition and reinforcement. Our findings suggest that destabilizing these ‘re-functional’ set points is a critical factor in the potential therapeutic benefits of psychedelic therapy, as well as the associated risks if misused or applied incorrectly.

## Shaking up the brain: how psychedelics act as neurological destabilizers

The treatment effect of psychedelics has often been described in reference to the destabilization or disruption of globally distributed brain dynamics. Empirically, this idea is inspired by findings showing that the psychedelic experience coincides with an increase of entropy in properties of spontaneous brain activity (Carhart-Harris et al. 2014, Carhart-Harris 2018), an effect that is likely to be most pronounced in the cortex (Bergström 1969, Kuang et al. 2021). Entropy, in its purest informational sense, is a dimensionless metric that captures the unpredictability or randomness of a dynamical phenomenon such as a time series.

Applied to the brain, an increase in entropy may indicate that neuronal circuits are exploring a wider array of patterns of activity, with potential departures from the ‘normal’ repertoire of states. The principle that brain entropy increases under psychedelics has been supported by a large number of empirical findings (Alonso et al. 2015, Lebedev et al. 2016, Liechti 2017, Schartner et al. 2017, Viol et al. 2017, Lyons and Carhart-Harris 2018, Timmermann et al. 2019, Herzog et al. 2020, Varley et al. 2020, Jobst et al. 2021, Luppi et al. 2021, Savino and Nichols 2021, Toker et al. 2022), and moreover, the principle that brain entropy tracks a principal dimension of conscious experience is increasingly well supported—now well beyond just research with psychedelics (John 2002, Carhart-Harris 2018, Keshmiri 2020). When we look at the brain as a hierarchical prediction engine—e.g. as one does under the so-called free-energy principle (FEP) (Friston 2010, 2013, 2019, Karl 2012, Parr et al. 2022), an increase in the entropy of spontaneous brain activity can be related to a decrease in the precision weighting—or the stability or reliability—of prior assumptions. Indeed, this is what is proposed in the so-called *RElaxed Beliefs Under pSychedelics* (REBUS) model (Carhart-Harris and Friston 2019) a unifying theory of the brain and psychological action of psychedelic compounds that bears relevance to their therapeutic potential. (The strengthened beliefs under psychedelics (SEBUS) model proposes that psychedelics enhance navigation abilities across belief landscapes, and this idea is not incompatible with the notion of a flattened landscape. A flattened landscape does not preclude the ability to navigate across belief landscapes; rather, it indicates that the system is not trapped in a fixed mode of interactions with interconnected systems. By engaging in a range of activities within a state space, the system can reduce free energy. Therefore, the formulation presented in this paper is consistent with the SEBUS model. Specifically, our argument suggests that the system is propelled out of a stuck state, thereby leading to greater navigation abilities across/through belief landscapes.)

According to the FEP and the ‘active inference’ component that it subsumes, the brain and behaviour (self) organize or are volitionally organized to minimize surprising encounters in the world by maximizing the reach, reliability, and efficiency of its internal models and behavioural schemas, in a fashion that is consistent with Hamilton’s principle of ‘least action’ (Hanc and Taylor 2004). Building on this, the REBUS model proposes that the entropy-enhancing action of psychedelics works against this imperative—i.e. psychedelics flatten the dynamical global landscape of brain states (Cruzat et al. 2022, Vohryzek et al. 2022), allowing for an easier escape from local optima, including ones that may have become excessively reinforced in pathology, but also creating a basal sense of felt uncertainty as the content of consciousness becomes unpredictable and ‘enriched’ relative to normal waking consciousness (Pollan 2018). (Flattening the landscape refers to a process that involves temporarily increasing the free energy within a system. When a system is trapped in a local minimum with high uncertainty, it becomes unable to evolve further from that point, as it has reached the maximum level of free-energy minimization. To transition the system away from this stuck state, it is necessary to perturb the system with just the right amount of uncertainty, i.e. the flattening of the landscape leads to a temporary increase in uncertainty and free energy. This perturbation allows the system to ‘bounce back’ and move towards another attractor or even undergo a critical transition, which can signify significant life changes.) This principle has been supported by recent experimental work on the acute action of psychedelics—finding a flattening of the brain’s energy landscape (Singleton et al. 2021), as well as the collapse of the brain’s principal hierarchical gradient (Girn et al. 2022), and the disruption of processes of Bayesian inference (Rajpal et al. 2022). Electroencephalography work has also substantiated the close relationship between brain entropy and the felt ‘richness’ of conscious experience (Tófoli and de Araujo 2016, Scott and Carhart-Harris 2019, Timmermann et al. 2019).

In the domain of perception, the downregulation of precision weighting on priors—as postulated by the REBUS model—will have various predictable effects, such as causing the instability of otherwise stable top-down perceptions (Carhart-Harris and Friston 2019). One might also hypothesize that the so-called learning traps and associated cognitive *misadjustments* may be impacted by psychedelics in a potentially useful way (Gopnik 2020), potentially yielding performance benefits—similar to those that have been observed in children’s healthy learning flexibility (Arán Filippetti and Krumm 2020). Evidence of increased learning rate under lysergic acid diethylamide (Kanen et al. 2021) and subacute cognitive flexibility after psychedelics (Wießner et al. 2022) may be relevant in this regard. However, we wish to caution that performance may be vulnerable to generalized deficit confounds under psychedelics, meaning that any hypothesized performance enhancement is likely to be dose dependent (i.e. establishing the right dosage for a specific case), ‘topping out’ into nonspecific deficits above a certain dose range—e.g. see Bayne and Carter (2018) for evidence of decreased cognitive flexibility under the acute effects of a psychedelic.

Overall, the REBUS model integrates the entropic brain hypothesis and the FEP framework (Carhart-Harris and Friston 2019). Central to this integration is that the entropic brain and the FEP inter-relate in that they share the notion of entropy as a dimensionless measure of uncertainty that can be applied to the brain, mind and behaviour as complex dynamical phenomena. The REBUS model has been supported by a number of empirical findings. Firstly, there is now substantial evidence to support the entropy brain principle, in terms of increased brain entropy in relation to the psychedelic state (Carhart-Harris et al. 2014), and other flexible mental states such as rapid eye movement sleep (Lee et al. 2013) and dreaming (Nardelli et al. 2019), jazz improvisation (Simon 2005), and deep meditation (Li et al. 2011, Kumar et al. 2021)—and reduced brain entropy under states of reduced consciousness such as deep sleep (Kung et al. 2022), the anaesthetized state (Fuentes et al. 2022) and disorders of consciousness (Visani et al. 2022). More specific support for REBUS can be found in perceptual changes under psychedelics (Kaiser and Gold 1973), altered beliefs under (Safron 2020) and after psychedelics (Timmermann et al. 2021, McGovern et al. 2022), and other trait changes (Aday et al. 2021), as well as decreased top-down processing seen through such metrics as travelling waves (Alamia et al. 2020), dynamic causal modelling (Muthukumaraswamy et al. 2013), and transfer entropy (Vicente et al. 2011, Barnett et al. 2020, Wang et al. 2022) reduced hierarchical organization under psychedelics (Girn et al. 2022) and the flattening of the brain’s energy landscape under (Singleton et al. 2021) and after (Daws et al. 2022) psychedelics.

It is important to note a more recent account, the Lysergic Acid Diethylamide (ALBUS) model, which partially supports the REBUS model by proposing that psychedelics can enhance navigation abilities across belief landscapes. This concept is not necessarily contradictory to the notion of a flattened landscape (Safron 2020). However, ALBUS also suggests that psychedelics can both directly and indirectly reinforce or relax beliefs depending on the individual and the context. By using the complex systems approach in this paper, we can build upon the REBUS model by considering the ease of tipping in relation to the topology of the landscape. Additionally, we can expand on the ALBUS model by examining both the geometry of the landscape and the strength of tips as sources of control energy.

As mentioned earlier, we have briefly summarized how an increase in the entropy of spontaneous cortical activity during the psychedelic state can be understood from an empirical and computational framework. In this paper, we will leverage these dynamical aspects to model the effect that psychedelics have on the psychological domain using dynamical/CST.

## How psychedelics and psychosocial context shape mental health

There is growing evidence that psychedelics can have a positive impact on mental health, particularly when applied as psychedelic-assisted therapy (Reiff et al. 2020). There is also robust and reliable evidence that the subjective effects of psychedelics can be predictive of their enduring therapeutic effects (Yaden and Griffiths 2020, Lutkajtis 2021). Future work is required to test whether these subjective effects are paralleled by tightly coupled and equally predictive brain activity markers.

We also hypothesize that the entropy or complexity of spontaneous cortical activity, as such can be indexed by metrics such as the Lempel–Ziv complexity or other compressibility algorithms (Carhart-Harris 2018a), will emerge as valuable explanatory and predictive markers of action. We believe that such discoveries will lend support to a unified model of the potential therapeutic action of psychedelics that places emphasis on an acute entropic brain action and related relaxation of assumptions (i.e. the REBUS model)—yielding an opportunity for the revision of pathologically reinforced patterns of cognition and/or behaviour underpinning symptoms of psychiatric illness.

In the present work, in keeping with a biopsychosocial approach to medicine, we wish to give an appropriate level of attention to psychosocial factors relevant to the psychedelic experience. In this regard, we adopt a relational, context-sensitive, and dependent approach, whereby we see individual experiences as coloured by personal historical and sociocultural factors (Carhart‐Harris 2018b). This perspective is consistent with the philosophical psychology of Ludwig Wittgenstein and recent accounts of enactivism and ecological psychology (Wittgenstein 1953, Gibson 1978, Maturana and Varela 1987, Anscombe et al. 1991, Varela 1997, Hutto 2013, Hutto and Myin 2017) that aim to situate the cultural and personal significance of experiences, emotions, and behaviours. According to this view, psychological objects or events have no absolute truth conditions but rather exist in a web of relations or mutual interdependencies.

The goal and approach of many psychotherapies are not to make difficult memories or emotions ‘disappear’ but rather to place them in a perspective whereby they can be better contextualized and understood, e.g. with greater equanimity. The so-called third-wave psychotherapies such as acceptance and commitment therapy (ACT) and mindfulness-based cognitive therapy place particular emphasis on allowing for the expression and mindful awareness of negative feeling states, as a method for processing and, ideally, dissipating their influence.

It could be said that the aim of these and other psychotherapeutic approaches is to replace ‘unhealthy’ patterns of thinking and behaving with ‘healthier’ ones, where what determines the ‘health’ of a cognitive style or behaviour rests on its embeddedness within a complex network of largely consensual norms and values, e.g. that view drug addiction, suicidal intent, chronic under-eating, or compulsive hand-washing as dysfunctional in relation to these norms and values.

Conversational psychotherapy can seek to better align a patient’s cognition and behaviour with societal norms and values, but this process is slow and difficult and may be even more effectively achieved if the patient discovers their own desire for better alignment or ‘connectedness’ (Carhart-Harris et al. 2018a) through a transformative experience and an epistemic process of self-realization—as such can occur via psychedelic therapy (Reiff et al. 2020).

In the following section, we propose a conceptual framework to understand psychopathology by examining its connection to dynamical systems theory (DST) at a specific level of analysis. This approach aims to establish meaningful connections between the subjective experience of psychopathology (phenomenology) and the underlying dynamics of the brain. By adopting this approach, we seek to identify heuristic and conceptually satisfying equivalences between these two domains.

## Complexity science: essential terminology

**Figure 1:**
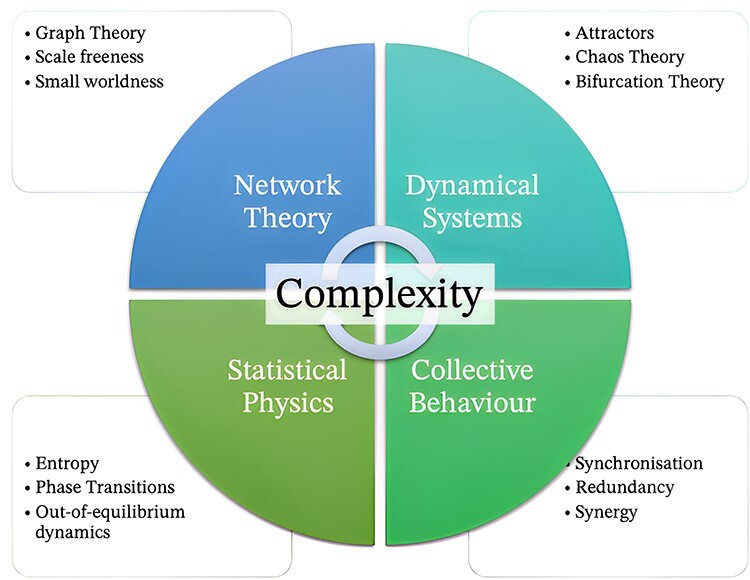
Complexity science is composed of an array of different techniques, which together aim to capture the unifying common features of the behaviour of multisystem interactions

Let us now introduce some fundamental ideas from complexity science, which will be instrumental in the development of our approach in the following sections. A complex system is a system whose behaviour results from a highly nontrivial aggregation of interactions, both between the parts of the system and also between the system and its environment (Mitchell 2009, Fieguth 2017, Thurner et al. 2018). Systems are complex to the extent that these relationships involve multiscales, complex external influences, nonlinearity, feedback loops, and multiplicity, which, in turn, may give rise to self-organization and emergent phenomena (Turkheimer et al. 2021). CST is an interdisciplinary research programme that aims to understand the underlying common features of complex systems that arise in different levels of nature—from physics, chemistry, and biology to sociology, economics, and art—by studying them with a range of tools including statistical physics, information theory, nonlinear dynamics, systems theory, self-organization, nonlinear systems, and network theory (Fig. 1). (CST is an interdisciplinary research programme that aims to understand the unifying features of complex systems that arise in different fields of science, including meteorology, sociology, economics, philosophy, psychology, and biology (Waldrop 1993). For this purpose, CST leverages methods of different disciplines including statistical physics, information theory, nonlinear dynamics, anthropology, and computer science.)

For reasons not yet entirely understood, complex physical systems and biological processes can self-organize without external direction or control, e.g. via an outside or centralized system (Karsenti 2008, Rosas et al. 2018, Kimura 2022).

This is a particular feature of complex living systems, and when it occurs, such systems attain states of ‘spontaneous’ order which seem to defy the second law of thermodynamics—i.e. the natural tendency for structures to degrade, dissipate, or die (Cilliers 2002, Gentili 2018). Self-organization typically occurs within complex nonequilibrium systems, i.e. systems that are not thermodynamically relaxed, but rather are subject to regular, complex interactions between and within their parts, and between the system and complex external influences, i.e. their ‘environments’ (Marzo Serugendo et al. 2003, Wolf and Holvoet 2004).

Theories of self-organization try to explain how different systems naturally tend towards distinctive types of intrinsic dynamical organization. One leading theoretical approach to self-organization is the notion of ‘self-organized criticality’, which studies how dynamical systems spontaneously tend towards distinct recurring patterns or states, i.e. attractors (Jensen 1998, Pruessner 2012, Bak 2013). As the name implies, self-organized criticality is often found close to a phase transition (or ‘critical point’ or zone) beyond which the system degrades into disorder or randomness (Bak et al. 1987), i.e. high entropy. Thus, critical systems can be viewed as exhibiting a functionally useful balance between self-maintenance and adaptability. In a poetic sense, they ‘surf’ uncertainty (Clark 2015) or entropy (Carhart‐Harris 2018b).

Complex systems can also be studied as dynamic processes, to the extent that a dynamical systems theory (DST) toolbox is used to make sense of transitions in their trajectories and patterns of behaviour. A system’s evolution can be mapped as a trajectory in time, which illustrates a system’s behaviour. The trajectory shows this behaviour as tending to fixed stable or semistable points, known as attractors, and avoiding fixed unstable points, known as repellers. The future state of a complex system is generated by its current state in the interaction with its external environment, which can be cast under a dynamic rule. A more complex system such as an adaptive system entails a more complex geometry of fixed points (Carmichael and Hadžikadić 2019).

Studying an adaptive system’s behaviour can help us to gain insight into its function, such as how the system adapts in relation to changing environmental circumstances. Studying this can also inform us about the system’s features or properties, such as its nonlinearities and interdependencies. The emergent complexity of a complex system arises precisely from the consequences of nonlinearities in the dynamics of the interacting subsystems, which are reflected in patterns of interactions among them at various timescales. One approach to conceptualize the complexity of a system is by how difficult it is to model a resulting behaviour (Crutchfield et al. 2009, Mitchell 2009). Modelling approaches that fail to acknowledge complexity—e.g. by reducing complexity to linear processes—will necessarily generate models that are not explanatory nor epistemically useful (Zeng et al. 2017). This is particularly important when planning interventions, e.g. a too simple model could cause us to intervene in a way that obtains an opposite effect from the one originally intended (Curtis 2016). This is crucially important for mental health interventions, as we develop in the following sections.

Sudden transitions can have a huge, potentially irreversible impact on a complex system—such as global changes in climate, shifts in ecosystems, crashing in financial markets, and sudden iatrogenic transitions in one’s psychological status, e.g., into a psychotic or depressive episode. Some sudden transitions can be described as ‘tippings’. These can occur when tiny changes to one or more of the system’s parameters lead to deep qualitative changes in the state of the global system. In nonresilient complex systems, tipping in one subsystem can result in tipping cascades and turbulence in the whole system (Carreras et al. 2002, Krönke et al. 2020, Klose et al. 2021, Sharpe and Lenton 2021). Interestingly, tipping and cascading may not necessarily have detrimental effects, e.g. if these phenomena can be planned for and harnessed for functional purposes, such as large-scale information transfer or functionally useful global system transformations (Brouwer and Carhart-Harris 2021).

The prediction of tipping can be useful for preventing, preparing for, or controlling their occurrence or consequences (Peng et al. 2019). Tipping can be induced in complex systems by intentional perturbations such as ‘temperature’ increases, causing bifurcations, fluctuations or noise near critical states, and rate-dependent variations of drift in control parameters of the dynamics (Ambika and Kurths 2021). Some of the points identified as critical for typing cascades include basins of attraction and fractality-induced tipping, as well as topological complexity of connections and interactions (Kaszás et al. 2019, Suchithra et al. 2020).

## Materials and method

Here, we apply a complexity theory approach to our understanding of psychopathology. As already stated, CST lends itself well to bridging phenomenology and psychopathology as it arguably approaches both at a functionally meaningful mechanistic level, where natural mappings between brain and experience can be made. We seek to bring CST to psychopathology by focusing on three main components: the *emergence from wholeness*, a *specific pattern or order*, and the notion of *stuck states*. To address the challenges of recovering from the latter, psychotherapeutic intervention can be thought of as aiding recovery via intentionally causing an initial and arguably vital destabilization. Indeed, empirical evidence for the value of destabilization in the therapeutic process already exists (Hayes et al. 2015, Olthof et al. 2020), and the theme of ‘sudden gains’ in psychotherapy is also relevant here (Shalom and Aderka 2020); notably, quantum change emphasizes the persisting consequences caused by the experience (Miller 2004, Cole-Turner 2022).

When studied from a complexity theory perspective, psychopathology can be seen as a specific type of dynamic pattern emerging from self-organizing interactions involving various biological, social, and psychological components of an adaptive system and its environment. According to our perspective, psychopathology cannot be properly understood by decoupling the individual from his/her environment. In this regard, it is crucial to take into account ALBUSs (Safron 2020) both strengthened and relaxed beliefs, which can enhance an individual’s connections to meanings and others in the world. By examining the relationship between an individual’s beliefs and their environment, we can gain a more comprehensive understanding of the factors that contribute to the development of psychopathology. Moreover, psychopathology is seen as an emergent phenomenon, in the sense that it depends on the coupling between the individual and his/her environment, and hence it cannot be reduced to the sum of the parts in isolation (Mediano et al. 2022).

Thus, we see psychopathology as a dynamic process, a self-organized pattern that arises from a specific type of interdependence between biopsychosocial components. This implies that psychopathology cannot be properly understood by isolating separate contributions of parts of the system but has to be understood and treated as a whole (Kelty-Stephen and Wallot 2017, van Geert and de Ruiter 2022). Understanding patients and psychopathology as complex ‘wholes’ means advancing an explanation that unifies the various scales that constitute the whole, which includes the psychological experience situated in a sociocultural setting.

Seeing psychopathology from the lens of complexity science also lends support to the controversial position that symptoms of mental illness should not necessarily be seen as reflective of a discrete *disorder per se*, rather they may be seen as reflective of a different *kind of order* (Olthof et al. 2020), i.e. an effective ‘next best’ order when, for quite logical and rational reasons, circumstances do not allow for a ‘healthy’ order, as it is generally understood. Here, we use ‘reorder’ and ‘reordering’ to refer to the abnormal but not necessarily ‘dysfunctional’ order that underlies symptoms of mental illness.

Thus, from this arguably more human and empathic perspective, psychopathology corresponds to a particular type of reordering that arises when specific patterns form within a complex dynamical process. Hence, while still being regarded as a mental health condition or disease, psychopathology is not seen as a disorder disrupting an otherwise healthy pattern—such as an imbalance in individual parts, i.e. neurotransmitters, hormones, or personality traits (DeYoung and Krueger 2018), but as a dynamical pattern itself. In other words, psychopathology is seen as a kind of order that is distinct from the dynamical patterns that we typically associate with the absence of a certain mental condition typically associated with health (Bosman 2017). [Notably, psychopathology can also be understood in terms of the absence of certain forms of order. This perspective provides a possible explanation for why the general factor of psychopathology, also known as the ‘P-factor’, appears to be inversely related to the meta-trait of Stability, which is a shared variance of conscientiousness, agreeableness, and inverse neuroticism. In terms of personality, the approach outlined in this article emphasizes the significance of increasing the meta-trait of ‘Plasticity’, which represents the shared variance of Openness and Extraversion and exists in a state of dynamic tension with Stability (Safron and DeYoung 2021)].

This dynamical view of psychopathology can be further developed following an enactive approach (Varela et al. 1992) operationalized via tools from DST. In this framework, individuals are seen as evolving coupled with their sociocultural environment, namely enacting their environment, which can be interpreted as a dynamical process whereby a system traverses a given ‘phase space’—i.e. the set of possible states or configurations available to the individual. In enacting their environments, the specific properties of how the individual is presently coupled with their environment result in the dynamical trajectory being more or less attracted to certain behaviours, represented by the system persisting on different regions of the phase space. DST then tells us that such *attractor sets* can be of different types: single stable points, periodic cycles, or the so-called strange attractors of complicated geometries (Ruelle and Isola 1989). Moreover, a given system can have multiple attractors and dynamically switch between them, which gives rise to metastable behaviour—which has been shown to be important for biological and social systems (Kelso 2012, 2021, Tadić and Melnik 2021).

The ways in which an individual navigates the geometry of its attractors determines its behaviour, which in turn feeds back into its environment and determines the future dynamical geometry that it will observe. This process can introduce bifurcations (i.e. the rupture of one attractor into two separate attractors) or more general dynamical phase transitions in which qualitative changes alter the attractor landscape, which characterize important processes of change in living systems (Sardanyés and Solé 2006).

**Figure 2:**
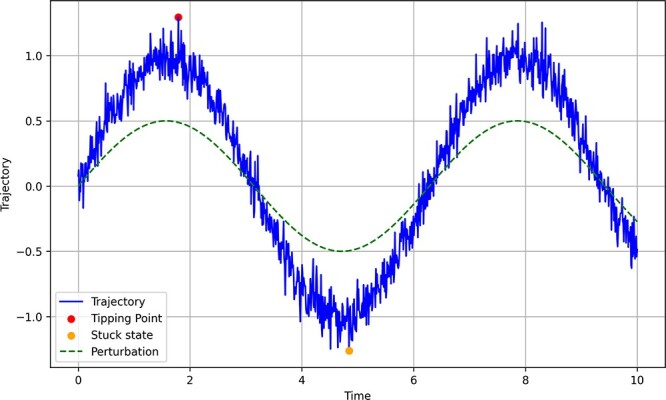
A depiction of how a system’s state (represented with a dot at time 4.8) evolves with a tendency towards certain ‘attracting’ points (basins of attarction or valleys) and avoiding repelleter (at time 1.8)

Emotional states themselves can be seen as attractors—e.g. sadness can be operationalized as a fixed-point attractor that, depending on how the individual is coupled with their environment, might exert more or less influence over an individual’s trajectories. Importantly, biological beings are a consequence of their actions, which implies that—all coupling conditions remaining equal—the more the system visits that attractor point, the more the system will be likely to visit it again, like running water carving out channels or canals (Waddington 1959). This process is consistent with experience or activity-dependent plasticity, Hebbian plasticity, and reinforcement learning. Individuals can also develop cycle attractors, e.g. becoming stuck in periodic shifts between being melancholic and euphoric—such as occurs in bipolar disorder (for illustration of these states as attractors, see Fig. 2).

Importantly, the same attractors can exert a stronger or weaker influence over a system, represented here by the depth of the valley—the deeper it is, the harder it is to go away from it and the easier it is to fall into it and become stuck. Psychedelics may flatten the attractor landscape (e.g. move from deep valleys in black to shallow valleys in blue), increasing the probability of the system’s state moving between attractors and hence promoting more flexible, adaptive, and metastable dynamics

Importantly, such experience-dependent reinforcement is the process by which attractors deepen, a process commensurate with increasing the precision weighting on priors if cast in a manner that is consistent with the FEP and its use of predictive coding and the Bayesian brain (Friston 2018). Here, the steepness of the sides of attractors or canals is directly proportional to the magnitude of the precision weighting of the relevant prior, substate, pattern, or attractor. Moreover, we argue that the process of carving out deep and abnormal attractors or priors can describe features of development and perpetuation of symptoms of mental illness, i.e. the brain and behaviour continue to adhere to the FEP (i.e. the minimization of uncertainty), but they do so via different, ‘abnormal’ strategies. While excessive canalization can be detrimental, a lack of stability can also be problematic for conditions such as schizophrenia. In addition to overly strong negative beliefs, insufficiently strong positive beliefs can also contribute to depression and anxiety. However, solely focusing on flexibility may overlook the importance of cultivating positive psychological factors and the potential risks of excessive destabilization from the unskilful use of psychedelics and certain forms of meditation. Self-organized criticality is characterized by a delicate balance of order and disorder, which is essential for the possibility of (generalized) evolution (Safron et al. 2022).

We acknowledge, however, that the term ‘abnormal’ could be questioned here given that certain ‘defensive’ strategies, e.g. internalizing in depression or escaping and relieving in addiction, are not uncommon.

Lastly, it is important to note that excessive chaos and high uncertainty, resulting in a flattened landscape, can have negative implications on the adaptation and mental well-being of a system. Mental health should not be associated with an overall general rule of fattening the landscape, which should be viewed simply as a therapeutic strategy. Rather, the key to avoiding psychopathology is achieving multistability. More precisely, systems must engage with the environment to avoid becoming isolated, as this creates a network of multistability that enables them to rebound from environmental stressors. Multistability refers to the ability of an individual’s psychological system to attain multiple stable states or attractors in response to changing environmental conditions. These attractors can correspond to various patterns of thoughts, emotions, behaviours, and beliefs. The ability to transition between these states is considered indicative of psychological flexibility and resilience. Thus, mental health is defined by the capacity to achieve and sustain a variety of stable states or attractors (multistability). This capability permits individuals to adjust to fluctuating circumstances, cope with stressors, and interact with the world in an adaptable and effective manner. Conversely, mental illness or psychopathology is the result of being confined to a rigid, single state (stuck state), unable to transition between states, leading to maladaptive thoughts, emotions, and behaviours.

Biological systems are always subject to external perturbation that cannot be predicted or controlled. It introduces small fluctuations in the system’s dynamics, nevertheless, attractor states represent favoured states that the system tends to stabilize into. In principle, this property of attractors imbues biological systems with favourable properties such as robustness or resilience, but it can become problematic when the attractor becomes too reinforced or rigid—e.g. aligning with cognitions or behaviours that clash with societal norms and values—such as it the case with most symptoms of psychiatric illness (Wichers et al. 2019). The paradox of stable attractor states is that they can provide an attractively low level of uncertainty, e.g. by finding solace in the effects of a drug of addiction or the apparent ‘safety’ of a ‘dark room’ of depression. However, a too-stable attractor or ‘stuck state’ affords reduced degrees of freedom, i.e. fewer possibilities for other forms of thought or action. It, therefore, affords a ‘narrower’ state of being. Here, we argue that this stereotypy—closely linked to excessive precision weighting on priors in a free-energy framework, or excessive experience-dependent plasticity in reinforcement learning—is the first component of mental illness. This is qualified to some extent by whether the relevant stereotypy is at odds with the norms and values of a given society or culture and by how pronounced this is, i.e. how reinforced or heavily (precision) weighted it is. These qualifiers should help to determine whether an ‘expert’ who has developed his/her experience-dependent expertise through extensive practice does or does not exhibit psychopathology. For example, they may not if their expertise is valued by a given culture or society, or if it is only moderately weighted.

An example of a common ‘stuck state’—that is often (but not always) regarded as pathological—is rumination. Rumination involves repetitive or stereotyped patterns of thinking that feature a pull towards a thematic core that is difficult to escape from. Although rumination begins with a desire to resolve a psychological issue—i.e. to examine, make sense, or learn from a social dilemma, major surprise, or trauma (Andrews and Thomson 2009)—it can easily turn into a psychopathological condition (as defined earlier). One can easily imagine a scenario where what might have started as an attempt to understand or resolve a past action/event (‘why didn’t I yell more loudly?’, ‘it was my fault for driving on the freeway in heavy rain’) leads to a stuck state (‘I’ll never get over this; I’ll never live a normal life again’). In depression, patients often continually ruminate about their failings, reiterate thoughts of guilt, and engage in self-critical inner narratives. In addiction, drug craving drives behaviour that is specific, narrow, and rigid; individuals with addiction ruminate on their preferred substance(s) of abuse: why they cannot get away from it, where they need to go, or what they need to do to get it and pay for it. In OCD and anorexia, there is excessive rumination about threats to the person, i.e. the effects of eating or overeating. As with addiction, ancillary behaviours such as paying for drugs via prostitution or binge purging in eating disorders can work to further reinforce the disorder through repeated and reinforced feelings such as shame. Similarly, although often inadvertently, interpersonal relations can shape around the illness, creating a psychosocial matrix of continued reinforcement, e.g. living among fellow drug addicts. All these factors and others will work to entrench canalized thought and behaviour (Waddington 1959).

Building upon the ideas exposed earlier, psychopathology can be better understood not by the nature of the beliefs supporting given thoughts or behaviours but by the *weight* they are given—in a free-energy sense, the problem lies more with the *precision weighting of priors* than whether they are inherently ‘false’ or not (Hesp and Hipólito 2022). (Excessive precision weighting can lead to the reinforcement of beliefs beyond a reasonable threshold, resulting in the generation of “false” or overly generalized conclusions.) Thus, in a CST sense, the problem lies not with what an attractor encodes but with the *depth* or *steepness of its attraction*, i.e. the relative difficulty (if not apparent ‘impossibility’) of moving away from the state. Said differently, the illness rests not with the content of specific states but with their inter-relations and dynamics. For example, it is reasonable and healthy that certain events should cause stress and/or anxiety, but healthy systems and individuals are able to bounce back from these stressors rather than become pulled into a web of mutually reinforcing thoughts, actions, and relations (Masten et al. 2021). According to our scheme, psychopathology is the excessive difficulty of moving away from a state or states, i.e. being in a stuck state that clashes with the norms and values of a given society or culture and does not allow for the psychological freedom that is the *sine qua non* of health.

Stuck states are closely related to how agents deal with uncertainty. Following the FEP (Friston 2013), one can operationalize surprise (although the psychological notion of surprise is distinct from the information theoretical formalization of it, in practice these two notions generally have good correspondence; information theory conceptualizes surprise simply as a measure of improbability) as the difference between an agent’s assumptions about the world and the world they actually encounter. Agents adapt to their environments in the ways they adjust to surprising events—including whether they can ‘bounce back’ from such surprises. Typically, as mandated by the FEP, one recalibrates from a surprising event by updating one’s assumptions or by altering one’s behaviour—or the world itself, such that surprises are less likely (Friston et al. 2012). However, agents do not simply find a dark corner and stay there—what is known as the ‘dark room’ problem, but minimize surprise within a longer, more far-sighted time horizon (Millidge et al. 2021), which usually leads them to act in specific ways. [‘A dark, empty room presents few surprises. The information reaching the eyes is constant, uniform, and unremarkable; effective soundproofing could do the same for the ears. Add some creative seating, and the whole experience will be as dull and predictable as any experience could be’ (Sun and Firestone 2020).] When confronted with a surprising event, an agent has at least two options: either (I) to tend towards a stuck state or (II) to reduce its surprise by adapting to the environment. An agent taking the former option, i.e. tending towards a ‘stuck state’, can be seen as potentially entering a phase transition towards a psychopathological situation, a *dark room*, or an oxbow lake forming apart from the main, freely flowing river.

Some environments lead to more surprising events than others; for example, people in the army often experience events with high levels of surprise while having few forms of action for reducing it, thereby adapting to the environment. From a psychopathology point of view, this means that it is likely that, faced with prolonged high levels of surprise, an agent will be ‘stuck’ in that state, even after being removed from that environment—a psychological condition that may be associated with PTSD.

Lastly, a third strategy for dealing with surprise is to tolerate a high amount of it. The so-called tolerance of uncertainty trait (Strout et al. 2018) is a useful construct in this regard, where a high tolerance of uncertainty may be conducive to resilience and thus good psychological health—as has been shown (Rettie and Daniels 2021). Here, one might surmise that the agent employs an especially broad time horizon for the minimization of free energy, i.e. by accepting a higher-than-average level of uncertainty they may more easily explore and thus broaden their models of the world and self, which they then ‘hold lightly’—as they recognize they may not apply absolutely.

One might connect this broad, patient, and flexible style of free-energy minimization and ‘light belief’ with Buddhist philosophy and practice as well as third-wave psychotherapies, such as ACT (Hayes 2019). Indeed, this explains the contention that Buddhist practice (Batchelor and Smith 2002) and third-wave cognitive behavioural therapies may be a particularly ‘good fit’ for psychedelic-assisted therapy (Fauvel et al. 2023), as well as the view that a combination of psychedelic drug administration and evidence-based psychological therapy is most beneficial, if not essential for reliable positive outcomes (Weston et al. 2020, p. 1261). Meditation practices are known for their effectiveness in cultivating compassion, acceptance, and self-awareness (Chilson 2018, Giraldi 2019, Hammer 2019, Tifft et al. 2022). With professional guidance, meditation may be a useful supplement to the psychological integration process (Craven 1989, Travis et al. 2018, Villamil et al. 2019). Indeed, as we shall discuss later, integration is considered by many to be a critical component of safe and effective psychedelic therapy (Carhart-Harris et al. 2018, Watts and Luoma 2020, Gründer and Jungaberle 2021), safeguarding against the risks associated with psychological destabilization and exploiting its potential benefits.

Taking inspiration from psychedelic therapy, here we argue that in order to confront the possibility of entering a stuck state rather than bouncing back from adversity or the actuality of having fallen into such a stuck state—and now needing to get out of it—a therapeutic intervention requires the induction of *destabilization. Destabilization* is hence defined as the induction of a dynamical phase transition involving either or both: (I) more energy or ‘temperature’ in the system to drive more random fluctuations—commensurate with higher entropy—or (II) the lowering of steep basins of attraction. We argue that the first process (i.e. a temperature or entropy increase) is the most accessible and manipulable, whereas the latter arguably requires more time, i.e. the longer the agent is away from an old attracting state or visiting others, the weaker the old attracting state will become—a process commensurate with extinction learning. We further argue this latter component may depend on the quality of integrative support and behavioural change after the psychedelic experience, e.g. removing or revising situational reinforcers—including interpersonal relationships that explicitly or inadvertently work to support the maladaptive habits. The ideal consequence of psychological destabilization and good subsequent integration is that a broader, more flexible global state space is promoted and sustained.

The process of destabilization may cause the enhancement of features of criticality (Chialvo 2010) as the global system moves out of a subcritical regime associated with ill health (Carhart-Harris 2018). Such features might include critical fluctuations, cascading, long-range correlations, scale-free or fractal dynamics, and critical slowing down (e.g. Kelso 1997). It is telling that many of these signatures of criticality have been observed in the brain under psychedelics (Luppi et al. 2020, Varley et al. 2020, Jobst et al. 2021, Toker et al. 2022). Here, we argue that the phenomenon of *destabilization* applies at social, psychological, and biological scales—as is the characteristic of CST mechanics. The next section expands on the notion of destabilization and explains how psychedelics act as destabilizers to promote mental health.

## Results

The next generation of medical interventions will arguably target stability and change (Olthof et al. 2020, Wichers et al. 2020). Broadly defined, *destabilization* has been a prominent concept in psychotherapy, often identified as a key mediator of psychological and clinical change (Mahoney 1991, Hayes et al. 2007, Bendit 2014, de Felice Msc 2014, Gelo and Salvatore 2016, Haynes et al. 2020). Notably, emphasizing active engagement with the world from an accepting and compassionate perspective can increase the impact of targeting stability and change.

Correspondingly, in CST, destabilization can be studied as a loss of pull from particular attractors, e.g. principally, by injecting noise, energy, excitation, or temperature into the system. In a free-energy scheme, the lowering of basins of attraction is commensurate with reducing their precision weighting—and thus, in a Bayesian formulation, the precision weighting of priors (Friston 2018). When this process occurs in a system that could be tuned closer to criticality (as opposed to over a tipping point into white noise—or pure randomness), such changes can give rise to interesting dynamical phenomena such as critical fluctuations and critical slowing down (Das and Green 2019, Olthof et al. 2020, Boers and Rypdal 2021)—which can be measured and quantified. Evidence shows that psychological destabilization, measured as increased fluctuations in psychological states, is a predictor of clinical change in both coded observational data of therapy sessions and repeated self-ratings (Hayes and Andrews 2020, Olthof et al. 2020). Such psychological destabilization can be interpreted as early indicators for upcoming clinical transitions (Scheffer et al., 2009 , Helmich et al. 2021, Dablander et al. 2022).

Destabilization could also be measured neurobiologically, e.g. via measures of brain entropy, diversity, or complexity (Breakspear et al. 2010, Nilsen et al. 2020, Sarasso et al. 2021). When destabilized, a system can gain more diversity in its dynamics or range of possible behaviours—i.e. it gains more degrees of freedom. This may have a number of interesting consequences if, e.g., destabilization tunes the system closer to criticality. For example, systems tuned closer to criticality often exhibit a longer recovery time after a given perturbation—a phenomenon known as ‘critical slowing’ (Hesse and Gross 2014) [phenomena such as critical slowing or susceptibility to catastrophic events can indicate the presence of hazardous forms of instability (Goekoop and de Kleijn 2021)]. One can draw parallels between critical slowing in CST and sensitivity to perturbation. In psychedelic therapy, greater sensitivity to perturbation could be related to the strongly hypothesized exaggerated influence of context on outcomes (Carhart-Harris and Friston 2019), some concrete examples of which include sensitivity to the therapeutic alliance (Murphy et al. 2022), suggestibility (Carhart-Harris et al. 2015), and the music played during the dosing session (Kaelen et al. 2018). If the contextual conditions are adverse, however, one can imagine how small perturbations could cause spiralling ruminative ‘loops’ that are difficult to break out of.

Another way we can view critical slowing is in relation to the ability to ‘bounce back’ from adverse conditions, where increased critical slowing would parallel a reduced ability to bounce back, or rather a greater sensitivity to perturbation. On a trait level, one might hypothesize that highly sensitive or susceptible individuals—or the so-called orchids—should be more susceptible to mental illness, a relationship for which there is much evidence (Homberg and Jagiellowicz 2022). This relationship may also bear relevance to the sometimes described ‘thin-skinned’ or ‘open-hearted’ quality of individuals in the aftermath of a psychedelic experience, where interpersonal support and a grounded psychosocial matrix (Eisner 1997) may be vital for a positive therapeutic trajectory [see Hayes and Andrews (2020) for relevant material]. Conversely, if the psychosocial matrix is adverse for such thin-skinned individuals, analogous to ‘returning to earth with a bump’—there could be a heightened risk of iatrogenesis. These insights could inform therapeutic approaches in psychedelic therapy, where, e.g., once the psychopathological attractor is destabilized, subsequent treatments targeted at supporting an alternative, healthier state space are promoted—a process also known as *integration* (Hayes et al. 2015, Schiepek et al. 2016).

This process may work best if participants are not too heavily primed with new belief systems and behavioural schemas. One can easily imagine how priming could go awry or raise ethical objections if not professionally managed. For example, objections could be raised regarding the manipulation of impressionable individuals via the pro-plasticity effects of psychedelics. This matter touches on old controversies in psychotherapy that could easily re-emerge in the context of psychedelic therapy if not pre-empted and safeguarded against. For example, the problem of ‘false memories’, the implanting or priming of false or questionable inferences and relational or sexual ‘boundary crossing’ are relevant here (Flom et al. 2003, Healy 2021)—and have already been reported in the context of psychedelic therapy. One way to tackle these risks is to address them in therapist training and to monitor preparation, dosing, and integration sessions to ensure good practice. Briefly, the ideal is to promote an open enquiring mindset, not one that is indoctrinated or manipulated in a specific way (Pilecki et al. 2021). Indeed, the latter could be seen as serving experience-dependent plasticity rather than the more generalized, nonspecific plasticity that is, arguably, the fundamental action of psychedelics.

This generalized plasticity is intimately related to the destabilization that we emphasize in this paper. This action can promote the breaking up of persistent, overly reinforced patterns of thinking and behaving, an action commensurate with ‘extinction learning’ (Glavonic et al. 2022). A light, undirected but empathetic approach from therapists is arguably the best way to avoid new reinforcements or even, in the worst of cases, retraumatization via the unprofessional repetition of boundary incursions. As seen in the section ‘How psychedelics and psychosocial context shape mental health’, the goal of psychotherapy is to weaken unhealthy patterns of thinking by bringing memories, feelings, thoughts, actions, and relationships into perspective, i.e. by nesting them in an extended context of mutual dependencies and relations that can then be slowly processed and understood. More traditional psychotherapeutic techniques and methods, as well as a spiritual practice, can help to achieve these aims, but the processes that lead up to such epistemic development can be greatly catalysed by the basic destabilizing action of psychedelics. The challenge for psychedelic therapy is to do this in a way that is sustainable and does not, e.g., involve the bypassing of a full maturational development (Kornfield 2001).

The basic entropic action of psychedelics is linked to a rich psychological experience that enables novel or diverse cognitive and affective perspectives. Richness and novelty are arguably the inverses of the narrow, canalized cognitive, and behavioural styles that characterize psychopathology. Subjective rating scales exist for measuring aspects of the psychedelic experience, such as the Emotional Breakthrough Inventory (see also Roseman et al. 2019), the Mystical Type Experience Questionnaire (Hood 1975, see also Siegel and Emmert-Aronson 2021), the Psychological Insight Questionnaire (Peill et al. 2022), the Challenging Experience Questionnaire (Barrett et al. 2016), and the Altered States of Consciousness scale (Studerus et al. 2010). There are also useful scales that are arguably best applied in the days after a psychedelic experience (the so-called afterglow’ period), such as the Psychological Insight Scale (Peill et al. 2022). Some researchers have also made use of simple single-item ratings, measuring such phenomena as the ‘richness of experience’ (D’Agostino et al. 2020), ‘ego-dissolution’ (Nour et al. 2016), and the intensity of complex and simple visual imagery (Carhart-Harris et al. 2016). Viewing these scales and the experiences they pertain to can enable us to understand dynamical processes of destabilization and the ‘positive’ and ‘negative’ psychological phenomena that parallel them, by which we mean phenomena that are gained or enhanced (i.e. positive) or lost or diminished (i.e. negative). Examples of positive or ‘gain’ phenomena include ‘insight’, ‘visions’, ‘challenging states’, ‘mystical type experiences’, and ‘emotional breakthroughs’—and examples of negative or ‘loss’ phenomena include ‘ego-dissolution’. The notion of ‘emotional breakthrough is becoming particularly relevant as a strong predictor of positive therapeutic outcomes (Aday et al. 2020, Roseman et al. 2019, Nutt et al. 2020, Spriggs et al. 2021, Kuc et al. 2022, Peill et al. 2022).

It is important to emphasize that specific experiences should not be viewed as reliable effects of a direct drug action but rather experiences whose likelihood of occurring is increased by a direct drug action but that critically depend on contextual influences (Wolf and Hopko 2008, Carhart-Harris et al. 2018). The popularized term ‘set and setting’ is often used in relation to psychedelic experiences (Leary et al., 2017), with ‘set’ referring to the preparation of the individual (including his/her personality structure, expectations, and mood at the time), while ‘setting’ accounts for (physical, psychological, and sociocultural) the characteristics of the environment in which the experience takes place (Schneegans and Schöner 2008, Schilhab and Esbensen 2019).

Overall, in this section, we have cast the destabilizing action of psychedelic drugs in the context of psychedelic therapy. Leveraging principles from complexity science, we explored the idea that psychotherapy may trigger clinical change by destabilizing unhealthy patterns of thinking and that psychedelics can be strong candidates for catalysing this. The widening of a self-centred point of view is ideally paralleled by a generalized sense of connectedness, tenderness, and peace that enables an unhurried reprocessing of one’s past, present, and future that is practised and realized (Peill et al. 2022).

## Discussion

We propose that incorporating insights and methods from dynamically CST, adaptive networks, and the FEP framework (Friston 2019, Hipolito 2019, Parr and Friston 2019, Da Costa et al. 2020) can enhance our understanding of the cognitive neuroscience of conscious experience and the basic and therapeutic action of psychedelic compounds. In contrast to eliminativist or excessively brain-centric approaches to consciousness and the psychedelic experience, we believe that CST offers an approach that respects the phenomenology of lived experience and acknowledges the relevance of emergent and dynamical phenomena in psychology, mental health, and psychedelic therapy. By incorporating insights from CST, we can gain a deeper appreciation of the complexity and nonlinear dynamics underlying the psychedelic experience and the therapeutic mechanisms that underlie its efficacy. This may ultimately inform the development of more effective and personalized psychedelic-assisted therapies for a range of mental health conditions.

Applying CST to psychedelic therapy for common mental health issues enables us to explain their action under a unified framework. One popular interpretation of psychedelic action is the REBUS model (Carhart-Harris and Friston 2019), which suggests that psychedelics work by relaxing high-level priors, allowing for new perspectives and interpretations of oneself and the environment. The REBUS model integrates the entropic brain hypothesis and the FEP framework, proposing that the entropy-enhancing action of psychedelics flattens the mind and brain’s dynamical landscape, thereby increasing uncertainty, allowing the global system to escape from overly reinforced local optima (e.g. associated with symptoms of mental illness) and explore a more diverse and balanced global state space over time. In this way, healthier ways of thinking and behaving can be cultivated.

The destabilizing effects of psychedelics are closely tied to their ability to increase entropy and relax beliefs, which in turn corresponds to their potential to dismantle heavily reinforced attractors or rigid patterns of thought and behaviour that underlie symptoms of mental illness. By incorporating a complex systems perspective into our understanding of psychedelic therapy, we aim to provide a more comprehensive account of how the mind and brain can be reconfigured through the destabilizing effects of psychedelics, as well as other interventions that share this fundamental property.

Taking a CST approach to understanding the REBUS model offers a framework to explore the relationship between a range of psychological constructs, including destabilization, uncertainty, conscious experience, beliefs, assumptions, habits, belief relaxation, precision weighting, confidence, learning, reinforcement, de-weighting, emotional breakthrough, insight, integration, and more. This approach not only helps us to better understand the psychological effects of psychedelic therapy but also provides a foundation for investigating their neurobiological underpinnings through techniques such as brain imaging.

One interesting offshoot of our application of CST to psychedelics is what it implies about the nature of psychological health. That is, according to our model, health may be defined by the depth of inter-relations and free-flowing exchange between different patterns of mind, brain, and behaviour, where, e.g., no one pattern is too dominant or weighted. Thus, healthier global patterns or dynamics should feature the dynamic nesting of psychological states (and the brain states they relate to) in an extended set of harmonious patterns that share the information they encode. This general rule of deep nesting and information exchange relating to healthier and happier feeling states should apply within the brain, as well as between the brain and body and between the body and other living beings and systems. For example, the rhythms and dynamics of these various systems and their subsystems should ideally inter-relate in a mathematically logical way, as is the case, e.g., with harmonics in sound and music. Empirical research demonstrates that brain waves in specific frequency bands align with distinct cognitive states (Deco et al. 2017, Basar 2022, Watanabe et al. 2023), which has been described as a resonance music chamber (Cabral et al. 2023).

The presented model is admittedly lacking in detailed explanations, yet it is believed to hold considerable intuitive appeal and may aid in understanding the unitive or nondual experiences associated with psychedelics and certain meditative states. The sense of interconnectedness that can arise within and after psychedelic experiences is also relevant, and the ‘symmetry theory of valence’ (Hesp et al. 2021, Smith et al. 2021) and the notion that self-organized criticality within and between systems may relate to positively valenced feeling states (Carhart-Harris et al. 2014) provide relevant frameworks to consider. We believe that this model deserves dedicated empirical investigation, particularly given the current lack of comprehensive knowledge in cognitive neuroscience about the biology of valence-specific emotion (Lindquist et al. 2016) and the potential benefits a better understanding could bring to mental health research and treatment.

## Conclusion

In conclusion, our CST-based approach suggests that psychedelics act as destabilizers, creating the conditions for the dismantling of overly reinforced set points or attractors that underlie symptoms of mental illness. The initial entropic and destabilizing drug effect can lead to a topological reconfiguration of the global energy landscape of the mind and brain. This effect can be harnessed by combining psychedelics with appropriate psychological support, including postdosing integration. Our approach offers new insights into the dynamic profile of psychological health and has the potential to inspire new investigations and approaches to mental health research and care. While there is growing evidence for the therapeutic value of psychedelic therapy, a better understanding of how it achieves positive therapeutic results is needed.
